# Melatonin and Kidney Health: From Fetal Stage to Later Life

**DOI:** 10.3390/ijms24098105

**Published:** 2023-04-30

**Authors:** Kuo-Shu Tang, Chun-Yi Ho, Chien-Ning Hsu, You-Lin Tain

**Affiliations:** 1 Department of Pediatrics, Kaohsiung Chang Gung Memorial Hospital, Kaohsiung 833, Taiwan; 2 Department of Pediatrics, Kaohsiung Municipal Feng Shan Hospital—Under the Management of Chang Gung Medical Foundation, Kaohsiung 830, Taiwan; 3 Department of Pharmacy, Kaohsiung Chang Gung Memorial Hospital, Kaohsiung 833, Taiwan; 4 School of Pharmacy, Kaohsiung Medical University, Kaohsiung 807, Taiwan; 5 Institute for Translational Research in Biomedicine, Kaohsiung Chang Gung Memorial Hospital, Kaohsiung 833, Taiwan; 6 College of Medicine, Chang Gung University, Taoyuan 333, Taiwan

**Keywords:** antioxidant, circadian rhythm, developmental origin of health and disease (DOHaD), melatonin, developmental programming, kidney disease, pregnancy

## Abstract

Melatonin, an endogenous hormone mainly released at night by the pineal gland, has multifaceted biofunctions. Emerging evidence points to melatonin having a crucial role in kidney health and disease. As the prevalence of chronic kidney disease (CKD) is still rising, a superior strategy to advance global kidney health is needed to not just treat CKD, but prevent it early on. Adult kidney disease can have its origins in early life. This review aims to evaluate the recent literature regarding melatonin’s effect on kidney development, its clinical uses in the early stage of life, animal models documenting preventive applications of melatonin on offspring’s kidney-related disease, and a thorough summary of therapeutic considerations concerning melatonin supplementation.

## 1. Introduction

Melatonin is a pleiotropic hormone mainly secreted at night by the pineal gland, which regulates the circadian rhythm [1]. In addition to its chronobiotic action, melatonin has antioxidant, anti-inflammatory, anti-carcinogenic, anti-apoptotic, anti-hypertensive and immunoregulatory properties [2,3,4,5]. Melatonin has a crucial role in human health and disease across the life span. Melatonin appears to be involved in normal pregnancy and fetal development [6,7]. The physiological effects of melatonin are various, and include multi-organ targets [2,3,4,5]. As a result, accumulating evidence supports the idea that melatonin holds promise in the treatment of various diseases, not only in adults but also in children and neonates [8,9,10,11].

Chronic kidney disease (CKD) is the main cause of death and disease worldwide [12]; it currently affects about 10% of the world’s population. Adult kidney disease can originate in early life, known as “Developmental Origin of Health and Disease” (DOHaD) [13]. Recent advances in human and animal studies have offered ample evidence that adverse environmental stimuli during kidney development increases the risk of CKD in adulthood via renal programming [14,15]. On the other hand, kidney disease can be averted in the early stage of life by reprogramming [16]. This vision suggests that the utmost attention is obligatory for global kidney health strategy, principally emphasizing the prevention of CKD at the earliest stage of life, not simply the treatment of established kidney disease [17]. 

**Figure 1:**
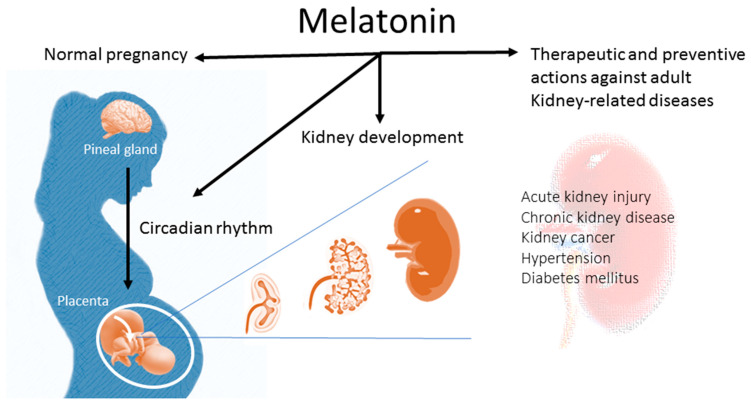
Schematic diagram highlighting the impact of melatonin in normal pregnancy and kidney development. Additionally, the use of melatonin in early life can protect against renal programming-induced adulthood kidney diseases later in life.

Although multiple positive actions of melatonin have been described, there is little known about the influence of melatonin on kidney health. This review, therefore, highlights the impact of melatonin on kidney development and opens important perspectives for the use of melatonin in preventive and therapeutic applications in kidney-related diseases later in life (Figure 1).

A literature review was carried out by searching the databases Embase, MEDLINE, and Cochrane Library using keywords relevant to melatonin, circadian rhythm, pregnancy, lactation, kidney disease, developmental programming, and DOHaD. A specific focus was put on the use of melatonin during pregnancy, infant, and childhood stages. The reference lists of articles were also examined to identify any additional references that would be related to this review.

## 2. Effects of Melatonin

### 2.1. Synthesis, Metabolism, and Action of Melatonin

Melatonin, or 5 methoxy-N-acetyltryptamine, was isolated from the bovine pineal gland and discovered nearly 60 years ago [18]. Melatonin is largely synthesized by the pinealocytes from tryptophan through hydroxylation, decarboxylation, acetylation, and methylation [19]. Melatonin synthesis and secretion is enhanced by darkness and inhibited by light [19]. Once secreted from the pineal gland, melatonin is quickly released into the systemic circulation to reach peripheral target tissues. Other than the pineal gland, many organs can produce melatonin, including the gastrointestinal tract, skin, retina, and bone marrow [20,21,22,23]. Take skin, for example; intracutaneous melatonin metabolites can form a potent anti-oxidative cascade through rapid local metabolism in an MT receptor-independent manner [23]. Therefore, endogenous melatonin production in skin may represent an anti-oxidative system to neutralize pathological changes such as skin aging and cancerogenesis [23].

The half-life of melatonin has been calculated to be around 30–60 min [24]. In the circulation, 70% of melatonin is bound to albumin, while the remaining 30% diffuses to neighboring tissues [4]. Melatonin is mostly metabolized in the liver and kidneys by P450 monooxygenases, and its main urinary metabolite is 6-sulfatoxymelatonin [25]. Only a small percentage (<5%) of blood melatonin is unmetabolized and excreted into the urine.

Melatonin acts through melatonin receptor-1 (MT1) and -2 (MT2), which are G protein-coupled receptors [4]. Melatonin receptors are widely distributed in the body, including the kidney [26]. In the kidneys, MT1 and MT2 receptors are predominately expressed in the kidney membrane area and basolateral membranes [26]. Both melatonin receptors have been shown to activate several signaling pathways, such as the ERK1/2 and the PI3K/AKT pathways [27,28]. Although melatonin regulates circadian rhythms via MT1 and MT2 receptors, the underlying mechanisms are not yet entirely understood and may differ between various tissues [29].

Additionally, the nuclear receptor retinoid acid receptor (ROR) has been suggested to mediate the genomic actions of melatonin [4]. However, recent work revealed that melatonin indirectly rather than directly regulates ROR activity [30]. Additionally, crystallographic evidence does not support the view that ROR is a nuclear receptor of melatonin [31]. Thus, whether ROR is a nuclear receptor of melatonin remains controversial [30]. Moreover, melatonin has receptor-independent effects. For example, melatonin-derived metabolites N1-acetyl-5-methoxykynuramine and N1-acetyl-N2-formyl-5-methoxykynuramine can act as powerful antioxidants [25]. Some melatonin actions could be secondary to its rapid metabolism to different metabolites [2,20].

While the physiologic functions of melatonin have been documented in multiple organ systems, its effects on the kidney are less well recognized. The peripheral circadian clock within the kidneys participates in various physiological functions, including glomerular filtration, tubulo-glomerular feedback mechanisms, the urine concentrating mechanism, circadian blood pressure (BP) rhythm, and the regulation of sodium transport [32]. Renal function is known to vary diurnally in healthy individuals. A previous study demonstrated that the daytime administration of melatonin to hamsters decreased urinary sodium and potassium concentrations as well as urine osmolality [33]. Since the rhythms of melatonin provide synchronization signals for peripheral clocks, dysregulated circadian rhythm and melatonin signaling may be related to kidney-related diseases [34]. These findings suggest that melatonin possibly impacts renal function by regulating proximal tubular function. Additional research is required to clarify the biochemical and physiologic details of melatonin in the kidneys so as to harness its therapeutic potential for kidney diseases.

### 2.2. Melatonin in Gestation and Fetal Development

Pregnant women have higher nighttime blood melatonin concentrations than non-pregnant women throughout gestation, reaching the highest level at term and falling to the basal level postpartum [35]. As shown in Figure 1, maternal melatonin is able to transfer across the placenta to the fetus, providing photoperiodic information to the fetus [36,37]. In addition to the pineal gland, the placenta can also secrete melatonin [38]. The difference is that the placenta does not produce melatonin in a circadian fashion, and it acts as a paracrine, autocrine, and endocrine hormone [38]. In the placenta, villous trophoblasts do not merely produce melatonin, but express melatonin receptors as well [38]. Melatonin in the placenta is able to work together with the MT1 and MT2 receptors to scavenge free radicals, consequently reducing oxidative damage in compromised pregnancies [39]. 

Melatonin receptors are widespread in the fetus from the early stages. In rodents, melatonin-binding sites in the pituitary gland are present in 15-day-old fetuses [40]. In the fetal human brain, melatonin receptors exist in many areas [41]. These data suggest that maternal melatonin has a role in the early stages of fetal development. Prior work revealed that a disrupted maternal and embryonic molecular clock impaired organogenesis in the fetus [42]. Another study showed that maternal melatonin deficiency caused circadian rhythm disruption and intrauterine growth retardation (IUGR) in adult rat offspring, which was prevented by maternal melatonin treatment [43]. The findings above support the fact that maternal melatonin is closely linked to fetal development as well as organogenesis.

## 3. Impact of Melatonin on Kidney Development

### 3.1. Kidney Development

Human kidney development begins as early as week three, followed by metanephros at five weeks of gestation [44]. Nephrogenesis is initiated when reciprocal interactions between the ureteric bud (UB) and the metanephric mesenchyme (MM) form the UB-derived collecting system and MM-derived nephron. The nephron is the basic structural and functional unit of the kidney [45]. It is composed of the glomerulus and the renal tubule. In humans, the first nephron is formed at nine weeks of gestation. A rapid increase in nephrons occurs from 18 to 32 weeks, and nephrogenesis is complete by 36 weeks of gestation. As nephrogenesis is not completed by preterm birth, nephron under-endowment is prone to be present in premature infants.

Apart from reduced nephron number, impaired nephrogenesis might cause a broad spectrum of malformed kidneys, namely congenital anomalies of the kidney and urinary tract (CAKUT) [46]. It is known that CAKUT is the major cause of pediatric CKD [46]. A low nephron number induces glomerular hyperfiltration and compensatory glomerular hypertrophy, thereby resulting in a vicious cycle of further nephron loss [45].

A previous study demonstrated that either global or local fetal clock disruption results in phenotypic defects that mimic CAKUT [47]. Our former work indicates that there are some common molecular mechanisms behind programming processes in various animal models [48].

### 3.2. Effects of Maternal Melatonin on Offspring Kidney

Maternal melatonin deficiency induces offspring hypertension, a common comorbidity of CKD [49]. There are several ways to induce maternal chronodisruption, including constant light exposure, diurnal light deficiency, continuous darkness, and photoperiod shifts [50]. One study showed that photoperiod shifts during pregnancy adversely program not only BP but also kidney function in adult rat offspring [51]. Another report demonstrated that the exposure of the mother rats to continuous light during pregnancy and lactation caused offspring hypertension [50]. Continuous light exposure alters several kidney genes responsible for high BP [52].

Using whole-genome RNA next-generation sequencing (NGS), we earlier analyzed the renal transcriptome from male rat offspring born to dams that received melatonin supplementation [53]. Melatonin (0.01% in drinking water) was administered during pregnancy and lactation to cover the entire period of nephrogenesis. At 1, 12, and 16 weeks of age, 455, 230, and 132 differentially expressed genes were identified in offspring’s kidneys, respectively. It looks like alterations of transcriptome are induced by melatonin declining over time. 

In support of melatonin’s epigenetic actions [54], maternal melatonin therapy can up-regulate several epigenetic regulators during kidney development [53]. Additionally, numerous biological pathways are regulated by melatonin administration during nephrogenesis [53]. Noteworthily, the tryptophan metabolism pathway is regulated by maternal melatonin supplementation in 1-week-old offspring’s kidneys as melatonin is derived from tryptophan. Several genes involved in the melatonin biosynthesis (i.e., *Tph1*, *Ddc*, and *Asmt*) and MT receptors (i.e., *Mtnr1b*, *Rora*, and *Rorb*) were up-regulated, suggesting that melatonin administration to mother rats can program metabolic and signaling pathways of melatonin in their offspring’s kidneys [53]. The findings above indicate that maternal melatonin administration is able to program the offspring’s kidney via the regulation of specific genes and pathways.

## 4. Preventive and Therapeutic Benefits of Melatonin

Through its pleiotropic effects, melatonin may be efficacious in managing various kidney-related diseases, such as hypertension [55], diabetes mellitus [56], acute kidney injury [57], CKD [58], and kidney cancer [59]. Taking CKD as an example, the renoprotective mechanisms of melatonin cover antioxidant, anti-apoptotic, anti-fibrotic, and anti-inflammatory effects [58]. Although plenty of preclinical studies have been conducted in this regard, evidence is scarce in the clinical setting. In view of the recent advances in DOHaD research, it has become evident that adulthood kidney-related diseases can be averted in the earliest stage by reprogramming [16]. Here, as the scope of the current review, we primarily focused on the use of melatonin from fetal to childhood stages as a therapeutic strategy for kidney disease. For more in-depth information regarding adulthood kidney disease, please refer to reviews published elsewhere.

### 4.1. Melatonin Use in Humans

Melatonin is a commonly used dietary supplement in the United States [60], while it is a prescription-only drug in Canada, Japan, the European Union, and Australia. Oral melatonin supplementation in humans has a favorable safety profile, with the usual daily doses of melatonin being 2 to 10 mg in diverse populations [61]. 

However, little information is available regarding melatonin’s use and safety in pregnant or breastfeeding women on the basis of clinical trials. At present, pregnant and lactating women are not recommended to use melatonin considering a lack of human studies [61].

As reviewed elsewhere [10,11], the use of melatonin as a treatment option has been evaluated in neonatal diseases, including hypoxic–ischemic injury, periventricular leukomalacia, respiratory distress syndrome, bronchopulmonary dysplasia, and sepsis. In infants and children, melatonin has also been widely used for purposes including sleep disorders and seizure disorders, and also as an alternative sedative drug [10,11]. Nevertheless, no information exists with regard to kidney disease within the pediatric population. Considering melatonin treatment gives useful results in adult kidney-related diseases, whether melatonin is an effective therapy in pediatric kidney disease is worthy of further evaluation. 

### 4.2. Melatonin Use for the Early Origins of Kidney Diseases in Animal Models

So far, there have been several animal studies reporting that melatonin exogenous supplementation during gestation could be beneficial for both mother and fetus [62], while only a small proportion of them have focused on offspring outcomes [55]. Here, we list in Table 1 a summary of reports relevant to reprogramming effects of melatonin on kidney-related diseases in offspring [63,64,65,66,67,68,69,70,71,72,73,74,75,76,77,78]. The therapeutic duration is restricted solely to fetal to early childhood stages prior to disease onset. 

Although melatonin has been examined for developmental programming in several species [79,80], the most studied species in this regard are rats. As revealed in Table 1, reprogramming effects of melatonin treatment on rat offspring have been evaluated from aged 8 to 27 weeks. As one rat month is the same as three human years [81], reported renal effects can be translated to human stages from adolescents to young adults. As a result, there remains a lack of information regarding the long-term effects of melatonin on older adults. 

Table 1 illustrates various maternal insults that can induce the developmental programming of kidney-related disease in adult offspring, which can be averted by the early use of melatonin. These early life insults include gestational chronodisruption [63], maternal continuous light exposure [65], maternal caloric restriction [66], maternal N^G^-nitro-L-arginine-methyl ester (L-NAME) exposure [67], maternal high methyl-donor diet [68], maternal high-fructose diet [69], maternal high-fructose diet plus post-weaning high-salt diet [70], antenatal glucocorticoid exposure [71], and antenatal glucocorticoid administration plus post-weaning high-fat diet [72]. 

Maternal melatonin supplementation could be administered during gestation [73,74], lactation [74,75], or both periods [65,66,67,68,69,70,71,72,73]. Additionally, melatonin treatment during early childhood has been tested in the adenine-primed pediatric chronic kidney disease (CKD) model [76], and the young spontaneously hypertensive rat (SHR) model [77,78]. Table 1 shows that the renal effects of the early use of melatonin included improvement in hypertension, reduced nephron number, kidney function, and altered renal transcriptome.

**Figure 2:**
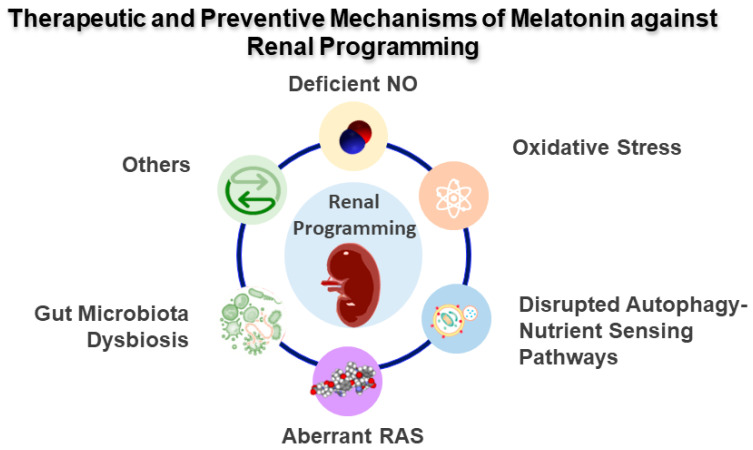
Therapeutic and preventive mechanisms of melatonin against renal programming. NO = nitric oxide; RAS = renin-angiotensin system.

Notably, certain mechanisms participate in the developmental programming of kidney-related diseases, such as deficient NO, oxidative stress, aberrant RAS, epigenetic regulation, disrupted autophagy–nutrient sensing pathway, glucocorticoid effect, and dysbiotic gut microbiota. Figure 2 is a graphic illustration of the therapeutic and protective mechanisms of melatonin interrelated to renal programming. Below, we will discuss each mechanism in turn.

### 4.3. Deficient NO

NO has an essential role in renal physiology and BP control [82]. Deficient NO has a crucial role in kidney disease and hypertension of developmental origins [83]. Conversely, adverse programing processes and renal outcomes can be averted by NO-targeting interventions during gestation and lactation. Melatonin can increase NO via reducing asymmetric dimethylarginine (ADMA), an inhibitor of NO synthase [84]. In young SHRs, the antihypertensive effect of melatonin coincided with the decrease of ADMA in the plasma and kidneys [77,78]. In a maternal NO deficiency model, deficient NO in gestation caused by L-NAME administration led to offspring hypertension in adulthood [67], whilst elevated BP and reduced renal NO could be restored concurrently by maternal melatonin therapy [67].

The perinatal use of melatonin is also beneficial to offspring hypertension relevant to the rebalancing of the ADMA-NO pathway in animal models of maternal caloric restriction [66], maternal high-fructose diet [69], and combined maternal high-fructose and post-weaning high-salt diets [70]. In an adenine-induced pediatric CKD model [76], melatonin therapy from 3 to 6 weeks of age prevented CKD-primed hypertension and kidney damage, which was associated with a reduction in ADMA. These data above revealed that melatonin could interact with NO, by which it provided beneficial effects against offspring kidney-related diseases.

### 4.4. Oxidative Stress

As a well-known antioxidant, melatonin displays a protective role against oxidative stress by scavenging free-radicals and activating antioxidant enzymes [2]. A previous review revealed that several environmental stimuli in pregnancy linked oxidative stress to the developmental programming of kidney disease [85], covering maternal nutrition imbalance, maternal disorders, environmental chemical and toxin exposure, and medication use.

Due to the low-antioxidant capacity of the fetus, a surplus of reactive oxygen species (ROS) under adverse intrauterine conditions overwhelms antioxidants, leading to oxidative damage and, thus, compromising fetal development [86].

Prior work provides evidence of how maternal melatonin therapy protected adult progeny against oxidative stress-related renal programming in models of maternal caloric restriction [66], maternal L-NAME exposure [67], maternal high methyl-donor diet [68], maternal high-fructose diet [69], and antenatal glucocorticoid exposure [71]. When targeting oxidative stress, the antioxidant actions of melatonin included decreased ROS-producing enzyme expression, reduced ROS production, increased antioxidant capacity, and decreased oxidative DNA damage.

Although melatonin has a significant impact on improving oxidative stress, one study revealed that maternal melatonin supplementation which averted the rise in BP in young SHR offspring might not be attributed to its antioxidant effects in kidneys [64]. Even though recent advances have been made in the understanding of how oxidative stress impacts renal programming, further work is needed to discover other protective mechanisms of melatonin, not just its antioxidant functions.

### 4.5. Disrupted Autophagy–Nutrient Sensing Pathways

Emerging evidence points to the dysregulation of nutrient sensing signaling and autophagy linking to a range of kidney diseases [87,88]. Several nutrient-sensing signals are involved in fetal programming [89], including silent information regulator T1 (SIRT1), peroxisome proliferator-activated receptors (PPARs), AMP-activated protein kinase (AMPK) and PPARγ co-activator 1α (PGC-1α). In pregnancy, maternal nutritional status influences fetal development via nutrient-sensing signals [89]. Melatonin can modulate autophagy by changing nutrient sensing pathways [90].

Melatonin supplementation protecting against kidney disease is connected to AMPK activation [91]. In a maternal methyl-donor diet model, offspring hypertension coincided with the reduced renal expression of SIRT1, AMPKα2, PPARβ, and PPARγ [68]. Another study explored perinatal melatonin therapy and found that its beneficial actions against high-fructose plus high-salt diet-induced offspring hypertension were related to regulating renal AMPKα2, AMPKβ2, SIRT1, SIRT4, PPARγ, and PGC-1α expression [70].

In an antenatal dexamethasone administration plus post-weaning high-fat diet model, the activation of genes related to nutrient sensing and autophagy prevented offspring’s hypertension [92]. These findings were consistent with a previous study showing that melatonin could mediate the renoprotective effect by upregulating the AMPK/SIRT1 axis and enhancing the autophagy in a rat model of diabetic nephropathy [93]. Although a link between melatonin and autophagy behind renal programming has been established, whether its reprogramming effect is attributed to the enhancement of autophagy needs to be evaluated further.

### 4.6. Aberrant RAS

Melatonin has a role in suppressing RAS [94], which is known as a hormonal cascade controlling BP and kidney development [95,96]. The angiotensin II (Ang II)/angiotensin-converting enzyme (ACE) cascade is known to be the classic RAS, which can be counter-regulated by the Ang (1–7)/ACE2 non-classic axis. In the developing kidney, RAS genes are vastly expressed and have a transient biphasic response with downregulation of the classic RAS in neonates that becomes normalized over time [97]. However, maternal insults enable the interruption of this normalization and improperly initiate the classic RAS, leading to kidney disease and hypertension in adult offspring [96,97].

The classic RAS axis is activated in melatonin-deficient hypertension [49]. BP-lowering effects have been reported in studies using melatonin in pregnancy and lactation, together with blocking the RAS, in animal models of gestational chronodisruption [63], maternal continuous light exposure [65], maternal caloric restriction [66], and maternal high-fructose diet [69]. Another study revealed that maternal melatonin therapy prevented offspring hypertension programmed by antenatal dexamethasone administration plus post-weaning high-fat diet, which coincided with an enhanced non-classic RAS axis by increasing renal *Agtr1b* and *Mas1* expression [72]. These observations above reveal a crosstalk between the RAS and melatonin behind renal programming, while additional research is necessary to confirm that the renoprotective actions of melatonin are directly RAS-dependent.

### 4.7. Gut Microbiota Dysbiosis

Another renoprotective mechanism of melatonin against renal programming might be due to its capacity to shape gut microbiota. The gut is a rich source of extrapineal melatonin with a ~400 times higher melatonin concentration in the gut than in the pineal gland [98]. Melatonin is a tryptophan-derived metabolite. Of note is that many tryptophan metabolites derived from gut microbiota participate in the developmental programming of kidney disease [99].

Emerging evidence supports gut microbiota dysbiosis in early life having adverse effects resulting in diseases in adulthood [100], such as kidney disease [101]. As reviewed elsewhere [101], a pathogenic interconnection, namely the gut–kidney axis, between the gut microbiota and kidney diseases is implicated in CKD and its comorbidities. Disturbed microbiota compositions and microbial metabolites are involved in pathogenesis. These metabolites include short chain fatty acids (SCFAs), trimethylamine-N-oxide (TMAO), tryptophan-derived uremic toxins, etc. [102,103,104,105].

A study investigating the effect of melatonin therapy for weeks in young CKD rats found that CKD-primed hypertension and kidney damage was prevented by melatonin [76]. The beneficial action of melatonin was accompanied by alterations in gut microbiota, including increased α-diversity, enhancement of the abundance of the phylum *Proteobacteria* and the genus *Roseburia*, and an improved TMAO metabolic pathway. Considering that several gut microbiota-targeted therapies have been applied for early prevention of CKD [101], a better understanding of how melatonin mediates gut microbiota underlying renal programming needs to be evaluated further.

### 4.8. Others

In view of the multifaceted actions of melatonin, there might be other mechanisms by which it provides an advantage: (1) by counteracting glucocorticoid programming, (2) by activating nuclear factor erythroid 2-related factor 2 (Nrf2), and (3) by regulating mitochondrial function. Similar to melatonin, glucocorticoid participates in the circadian rhythm [106]. Glucocorticoid and melatonin can downregulate each other’s receptors [49,107]. Considering that melatonin therapy prevented glucocorticoid programming-induced hypertension, the interplay between melatonin and glucocorticoid on renal programming deserves further elucidation. Additionally, melatonin can act like an Nrf2 activator [108]. In this regard, the coupling of melatonin and its metabolites to the activation of Nrf2 has been demonstrated in various organ systems [109,110,111,112]. Prior work indicated that Nrf2 activation has benefits for the developmental programming of hypertension [92,113]. Although this remains speculative, Nrf2 and other potential mechanisms are awaiting further clarification.

Melatonin can be specifically targeted to the mitochondria, where it acts as an antioxidant [114]. In mitochondria, cytochrome c is a natural scavenger of H_2_O_2_, preventing its accumulation. When electron transport is disrupted, the cytochrome c-dependent pseudo-peroxidase reaction with melatonin could become dominant to exhibit a protective mechanism [115]. Considering substantial evidence that has accumulated on the protective role of mitochondrial targeting against kidney disease [116], it is interesting to know how melatonin would coordinate mitochondrial interactions with the developing kidney to later determine kidney health and disease.

## 5. Pending Issues and Future Directions

If melatonin is be used as a potential intervention in pregnant women to prevent their offspring’s kidney disease, one major concern is its safety. A recent review revealed that a total of seven of the pregnancy studies and three of the lactation studies involved exogenous melatonin [117]. The dose of melatonin utilized in these studies ranged from 8 to 30 mg daily. No major adverse events or safety concerns were stated in most studies [117], except for one case of reported bleeding [118]. Nevertheless, currently there is a lack of clinical trials of melatonin use during pregnancy and lactation, especially trials related to offspring outcomes. According to the available evidence, no conclusions can be made about the safety of exogenously administered melatonin during gestation and lactation on the long-term outcome for the babies perinatally exposed.

Another pending question that needs to be addressed is the ideal dose and administration route. Most human studies using 10 mg of daily melatonin are not adequate to provide an adequate comparison with data on the therapeutic dose derived from animal studies [119]. The doses of melatonin used should be evaluated in the 40–100 mg/day range in view of the equivalent human doses of melatonin based on preclinical data. Notably, melatonin acts as a pro-oxidant at very high concentration (1–10 mM) [120]. In this situation, melatonin cannot be considered as a hormone but a context-dependent regulator. Considering that melatonin might display pro-oxidant activity when used at high concentrations [120], there is an utmost need for further research to recommend a maximum dose of melatonin in humans.

Melatonin is traditionally administered orally, while its drawback is low bioavailability due to fast release. Consequently, melatonin-sustained release formulations to humans via oral, intranasal, transdermal, and transmucosal administrations have been developed [121]. Nevertheless, whether these routes are suitable for pregnancy and whether melatonin’s pharmacokinetics are different in pregnant women are still not known [121].

Currently, melatonin might be the best peripheral biomarker for the circadian clock [122]. The onset of melatonin secretion under dim light conditions is the single most accurate marker for assessing the circadian pacemaker [122]. Accordingly, there have been several assays developed to analyze melatonin in blood and saliva for this aspect [123]. As its daytime physiological level is very low, there is a need for a specific assay for melatonin that is sensitive enough to detect low concentrations (<2 pg/mL). To date, several analytical methods for the quantitative measurement of melatonin concentrations in the plasma and saliva have been developed, including liquid chromatography with mass spectrometric detection (LC)–MS, gas chromatography (GC)–MS, radioimmunoassay (RIA), and enzyme-linked immunosorbent assay (ELISA) [123]. In clinical studies, ELISA and RIA are the most commonly used methods. However, many studies have been considered either meaningless or flawed due to extremely high levels caused by poor assay specificity [123]. Given that people who have no pineal gland display extremely low (<1 pg/mL) circulating levels of melatonin [124] and that melatonin level varies by age, with the lowest levels in young infants [125], future work in developing a simple high-precision method for determining melatonin in clinical practice is of utmost necessity.

Melatonin can be identified in both animal foods and edible plants [126]. A related question is whether dietary melatonin in pregnancy is appropriate for an efficient protection mechanism for offspring’s kidney disease The Mediterranean diet has been recommended for patients with CKD [127]. Part of this beneficial action might be attributed to the components of Mediterranean diet, which cover several melatonin-rich foods such as fish, red wine, olives, nuts, and fruits. Hence, some melatonin-rich foods might be of great value for development into functional foods, which would contribute to the prevention and treatment of kidney diseases.

As melatonin has pleiotropic biofunctions, its protective actions are difficult to predict or evaluate with a holistic approach in an experiment. Is there a dose-dependent mechanism behind the reprogramming effect of melatonin? Which protective mechanism might be most important? If so, what is the efficient dose and when should melatonin be implemented, and in which way, to mediate a specific protection mechanism? All these questions are still open-ended. Therefore, additional work in developing an ideal methodology is required to obtain a full-scope view of its protective mechanisms to ensure that melatonin therapy would only apply in the right direction.

## 6. Concluding Remarks and Perspectives

There is substantial evidence that melatonin participates in the pathophysiology of kidney health and disease. Although the use of melatonin as a potential preventive strategy is promising in preclinical studies, more work needs to be done to deliver it clinically.

So far, safety and efficacy data are largely lacking regarding the use of melatonin during gestation and breastfeeding and its reprogramming effects on offspring’s kidney disease. This review emphasizes the need for clinical studies in this aspect, including on exogenous melatonin, during pregnancy and lactation. Additionally, it is crucial to establish guidelines for the clinical use of melatonin for pregnant and lactating women.

In conclusion, melatonin contributes significantly to kidney health. Melatonin therapy in pregnancy and lactation can serve as a reprogramming strategy to prevent kidney disease while clinical translation is pending. Our review highlights a new path for the use of melatonin in working towards reducing the global burden of kidney disease.

## Figures and Tables

**Table 1:** Renoprotective effects of melatonin use in early life protect against early origins of kidney-related diseases in animal models.

Dose and Treatment Period	Animal Model	Species/ Gender	Age at Evaluation	Renoprotective Effects and Mechanisms	Ref.
Gestation					
2 μg/mL in drinking water from gestational day 0 to 18	Gestational chronodisruption	SD rat/F	90 days	Restored specific kidney genes related to BP control	[63]
10 mg/kg/day in drinking water	Genetic hypertension	SHR/M	8 weeks	Decreased the rise in BP	[64]
Gestation and lactation					
0.01% in drinking water	Maternal continuous light exposure	SD rat/M	12 weeks	Prevented hypertension and restored the aberrant RAS	[65]
0.01% in drinking water	Maternal caloric restriction	SD rat/M	12 weeks	Prevented hypertension, reduced oxidative stress, restored the aberrant RAS, and increased renal NO	[66]
0.01% in drinking water	Maternal L-NAME exposure	SD rat/M	12 weeks	Prevented hypertension, reduced oxidative stress, and increased renal NO	[67]
0.01% in drinking water	Maternal high methyl-donor diet	SD rat/M	12 weeks	Attenuated hypertension, reduced oxidative stress, and altered renal transcriptome	[68]
0.01% in drinking water	Maternal high-fructose diet	SD rat/M	12 weeks	Prevented hypertension, reduced oxidative stress, restored the aberrant RAS, and increased renal NO	[69]
0.01% in drinking water	Maternal high-fructose diet plus post-weaning high-salt diet	SD rat/M	12 weeks	Attenuated hypertension, restored NO system, and improved nutrient sensing signals	[70]
0.01% in drinking water	Prenatal dexamethasone exposure	SD rat/M	16 weeks	Prevented hypertension, increased nephron number, and reduced oxidative stress	[71]
0.01% in drinking water	Prenatal dexamethasone exposure plus post-weaning high-fat diet	SD rat/M	16 weeks	Prevented hypertension and restored the aberrant RAS	[72]
20 μg/mL in drinking water	Genetic hypertension	SHR/M	27 weeks	Prevented the rise in BP	[73]
Lactation					
0.01% in drinking water	Neonatal dexamethasone exposure	SD rat/M	16 weeks	Prevented hypertension and preserved histone deacetylase gene expression	[74]
0.01% in drinking water	Neonatal dexamethasone exposure	SD rat/M	16 weeks	Prevented hypertension, increased renal melatonin level, and upregulated MT2 protein expression	[75]
Early childhood					
10 mg/kg/day in drinking water from 3 to 6 weeks of age	Adenine-induced CKD	SD rat/M and F	9 weeks	Prevented hypertension, attenuated kidney injury, increased NO, and altered gut microbiota	[76]
0.01% in drinking water from 4 to 10 weeks of age	Genetic hypertension plus L-NAME exposure	SHR/M	10 weeks	Prevented hypertension, reduced renal oxidative stress and ADMA concentration	[77]
0.01% in drinking water from 4 to 12 weeks of age	Genetic hypertension	SHR/M	12 weeks	Prevented hypertension, reduced oxidative stress and plasma ADMA concentration	[78]

L-NAME = N^G^-nitro-l-arginine methyl ester; CKD = chronic kidney disease; SD = Sprague Dawley rat; SHR = spontaneously hypertensive rat; M = male; F = female; NO = nitric oxide; RAS = renin-angiotensin system; BP = blood pressure; ADMA = asymmetric dimethylarginine; MT2 = melatonin receptor 2.

## Data Availability

Data are contained within the article.
